# Towards Understanding the Pathogenicity of DROSHA Mutations in Oncohematology

**DOI:** 10.3390/cells10092357

**Published:** 2021-09-08

**Authors:** Dmitrii S. Bug, Artem V. Tishkov, Ivan S. Moiseev, Yuri B. Porozov, Natalia V. Petukhova

**Affiliations:** 1 Bioinformatics Research Center, Pavlov First Saint Petersburg Medical State University, 197022 St. Petersburg, Russia; dmitriybs@1spbgmu.ru (D.S.B.); tishkovav@1spbgmu.ru (A.V.T.); 2 R.M. Gorbacheva Scientific Research Institute of Pediatric Hematology and Transplantation, Pavlov First Saint Petersburg State Medical University, 197022 St. Petersburg, Russia; moisiv@mail.ru; 3 World-Class Research Center “Digital Biodesign and Personalized Healthcare”, I.M. Sechenov First Moscow State Medical University, 119991 Moscow, Russia; 4 Department of Computational Biology, Sirius University of Science and Technology, 354349 Sochi, Russia

**Keywords:** DROSHA, myelodysplastic syndrome, variants effect prediction, protein modeling, molecular dynamics

## Abstract

Myelodysplastic syndrome (MDS) refers to a heterogeneous group of closely related clonal hematopoietic disorders, which are characterized by accumulation of somatic mutations. The acquired mutation burden is suggested to define the pathway and consequent phenotype of the pathology. Recent studies have called attention to the role of miRNA biogenesis genes in MDS progression; in particular, the mutational pressure of the *DROSHA* gene was determined. Therefore, this highlights the importance of studying the impact of all collected missense mutations found within the *DROSHA* gene in oncohematology that might affect the functionality of the protein. In this study, the selected mutations were extensively examined by computational screening, and the most deleterious were subjected to a further molecular dynamic simulation in order to uncover the molecular mechanism of the structural damage to the protein altering its biological function. The most significant effect was found for variants I625K, L1047S, and H1170D, presumably affecting the endonuclease activity of DROSHA. Such alterations arisen during MDS progression should be taken into consideration as evoking certain clinical traits in the malignifying clonal evolution.

## 1. Introduction

Myelodysplastic syndrome (MDS) refers to a heterogeneous group of closely related clonal hematopoietic disorders commonly found in the aging population. All are associated with ineffective hematopoiesis with one or more peripheral blood cytopenias, and characterized by accumulation of somatic mutations [1] along with genome instability and high incidence of secondary cancerogenic genetic events, determining frequent transformation of MDS into acute myeloid leukemia (AML) [2]. The set of genetic alterations defines the disease prognosis and the choice of specific therapy. There is one hypothesis on MDS etiology stating that the mutations in mesenchymal stromal cells (MSCs) can disrupt the microenvironment, which triggers MDS initialization. In particular, the downregulated expression of both miRNA processing endonucleases, *DICER1* and *DROSHA*, was demonstrated in MSCs from myelodysplastic syndrome patients compared to normal cells [3]. This observation is accompanied by global miRNA dysregulation and consequent protein expression alteration during MDS progression [4]. A recent study confirmed downregulation of *DICER1* in MSCs derived from MDS patients and miRNA profile deregulation that in fact induces mutagenesis in hematopoietic stem cells via cellular cross-talk, leading to oncogenesis [5]. Moreover, selective deletion of the miRNA processing endonuclease DICER1 in murine mesenchymal osteoprogenitors induces markedly disordered hematopoiesis with several features of MDS, indicating the role of this gene in mesenchymal “stroma” as a primary regulator of tissue function [6]. A recent analysis of MDS clinical data revealed the high mutational burden in both miRNA processing genes and their association with common MDS mutations [7]. Taking these facts together, it can be concluded that the genes of miRNA biogenesis are under mutational pressure during cancer progression, and their disruption can alter cellular proliferation through miRNA regulation. Therefore, the investigation of mutations’ pathogenicity in the context of oncohematology might shed light on the functional importance of these proteins and the acquired mutations under tumor evolution. The present study is dedicated to analyzing *DROSHA* missense variants in order to evaluate their effect on protein structure and stability using different computational algorithms and molecular dynamics simulations. The significance of *DROSHA* mutations was not previously demonstrated as thoroughly as for *DICER1*—its major cooperative partner within the molecular pathway [8]: DROSHA converts pri-miRNAs into pre-miRNAs, performing a key step in miRNA maturation [9]. Considering the common function in the cell, the same pathological consequences can be supposed for *DROSHA* alterations: primary defect of *DROSHA* in osteoprogenitors might lead to an irrelevant microenvironment for hematopoesis. For that purpose, all mutations of this gene found to occur in hematological tissue were studied in detail to provide additional insights into the structural and functional role of DROSHA, and presumably consequent miRNA biogenesis alteration in oncohematology.

## 2. Materials and Methods

### 2.1. Data Collection: Selection of Variants

Since mutations in the coding region are likely to be the main disease-causing factor, they were selected for the analysis. The COSMIC (https://cancer.sanger.ac.uk/cosmic (accessed on 6 July 2021)) database was used to obtain variants of DROSHA protein reported in hematopoietic and lymphoid tissue; in sum, 13 coding nonsynonymous variants were collected (A27G [10], Q186*, P216 [11], R252Q [12], I387M, N420H [13], I625K [14], E727K [15], P808S [13], L1047S [16], H1170D [17], T1239S, I1164Qfs* [10]). Additionally, 3 variants were joined (P56S, R358Tfs*18, P356Lfs*121), acquired in MDS patients [7].

### 2.2. Sequence-Based Prediction of Deleterious Nonsynonymous Variants

In order to sort the most deleterious substitutions from tolerant ones, the functional context of missense mutations was predicted using the canonical sequence of DROSHA (isoform 1 Q9NRR4-1) by the default settings of different in silico prediction algorithms: SIFT [18], PolyPhen-2 [19], MutPred [20], PROVEAN [21], nsSNPAnalyzer [22], MAPP [23], PhD-SNP [24], SNAP [25], and PANTHER [26]. The MutationTaster tool was used to evaluate the consequence of frame-shifting mutations on the *DROSHA* transcript (Genbank ID NM_013235) [27]. The algorithms are described in Supplementary Table S1. Based on the consensus classifier pointing to the prioritized estimation in the PredictSNP tool [28], the most intolerant variants were selected for further analysis.

### 2.3. Structure-Based Prediction of Variants’ Effect on Protein Stability

The most deleterious variants selected after sequence-based prediction were further analyzed to evaluate the effect of mutations on thermodynamic stability using the following algorithms with default settings: mCSM [29], MUpro [30], i-MUTANT-Suite [31], SDM [32], DUET [33], PremPS [34], and Maestro [35]. The effect of mutations on DNA affinity was assessed using mCSM-NA [36] on mutant models with nucleic acid scaffold on the basis of PDB model 6V5B. These methods are described in Supplementary Table S1. It should be noted that each tool applies its own score and threshold to evaluate the mutation to be either destabilizing or stabilizing to the protein structure. Generally speaking, previous analyses of missense variations in different human diseases reported the stability boundaries without any immediate effect on protein functionality as 1–3 kcal/mol [37,38]. Otherwise, the mutations leading to a protein stability change of more than 2 kcal/mol were predicted as the most damaging and contributing to the most severe disease outcome [39]. Therefore, based on the previous studies, the absolute value of Gibbs energy change of more than 1.5 kcal/mol can be considered the threshold to evaluate the mutation effect on the protein stability in the present analysis: ΔΔG < −1.5 kcal/mol is highly destabilizing and ΔΔG > 1.5 kcal/mol is highly stabilizing. The sign of the predicted score depends on the tool selected (e.g., PremPS, Maestro, and FoldX4 use positive signs to indicate the destabilizing mutations).

### 2.4. Prediction of Changes in Vibrational Entropy and Normal Mode Analysis

In order to determine the effects of the mutations on the flexibility of the protein, the changes in vibrational entropy were predicted and normal mode analysis was performed. The FoldX4 was used [40]—an empirical force-field approach calculating free energy changes between native and mutant forms of the protein. In addition, ENCoM (an elastic network contact model) [41] was performed, which is a coarse-grain NMA method that considers the nature of the amino acids and aids in calculating vibrational entropy changes upon mutations. Additionally, DynaMut [42] and DynaMut2 [43] were also used as consensus predictors of protein stability based on the vibrational entropy changes predicted by ENCoM and the stability changes predicted by graph-based signatures that are used in the mCSM program.

### 2.5. Protein Structure Modeling

DROSHA 3D-structure PDB ID 6V5B [44] was selected from the Protein Data Bank (PDB) [45] as it contained the most complete protein sequence that was appropriate to study the specified mutations. The initial cryo-EM structure (resolution 3.70 Å) consisted of DROSHA region 411–1365 amino acids (aa) with both Zn^2+^ and Ca^2+^ ions, which were taken for further analysis. Besides the basic protein modeling alone, RNA was included into the DROSHA structure to study the stability of the complex and affinity to the ligand under the site-specific mutagenesis.

All stages of protein modeling and analytical calculations were performed using the Schrödinger molecular modeling suite (Schrödinger Suite 2020-4, Schrödinger, LLC, New York, NY, USA, 2020). Homology modeling in the Prime package [46] confirmed 6V5B as being the best hit, and additional loop 1356–1374 aa was built and refined by predefined options for smaller loops in Prime (VSGB salvation [47], OPLS3e force-field [48,49], subjob-protocol 1). Unfortunately, the N-terminal 410 aa region (neither of the main protein domains contain this disordered locus) was not reconstructed by Prime homology modeling due to its low-complexity nature and a lack of appropriate template. The final DROSHA model consisted of 411–1374 aa, and the loop 463–500 aa was not resolved as it represents a highly disordered protein region. The protein structures were prepared using the Protein Preparation Wizard (PPW, Schrödinger Suite 2020-4, Schrödinger, LLC, New York, NY, USA) [50]. No problems were reported in the processed protein structure.

### 2.6. Molecular Dynamics (MD) Simulations

MD simulations were performed using the Desmond package [51]. The MD system was set-up in “System Builder” in Maestro as follows: the TIP3P water model [52] was used to simulate water molecules, the buffer distance in the orthorhombic box was set-up at 10 Å, a recalculated amount of Na^+^/Cl^−^ ions were added to balance the system charge and were placed randomly to neutralize the solvated system, and additional salt was appended for a final concentration of 0.15 M in order to simulate physiological conditions.

Molecular dynamic simulations were conducted with the periodic boundary conditions in the NPT ensemble class using OPLS3e force-field parameters [48,49]. The temperature and pressure were kept at 300 K and 1 atmospheric pressure respectively, using Nosé-Hoover temperature coupling and isotropic scaling [53]. The model system was relaxed before simulations using Maestro’s default relaxation protocol, including two stages of minimization (restrained and unrestrained) followed by four stages of MD runs with gradually diminishing restraints. MD simulations were carried out by running for 50 or 200 ns, recording the trajectory configurations obtained at 50 ps intervals.

### 2.7. Protein Site-Specific Mutagenesis

First, the preprocessed and refined structure of wild-type DROSHA was relaxed by MD simulation for 50 ns in order to obtain the system with minimized energy. The recorded trajectories were clustered, and total energy values of the representative structures were calculated in Prime (selected parameters VSGV, OPLS3e). The structure with the minimal energy was employed in further long MD simulations and protein mutagenesis. Specific mutations were introduced into the structure by the 3D Builder Panel in Maestro, and side-chain rotamers were refined. The local structure around the inserted mutation was minimized. Prime side-chain prediction was carried out to find an appropriate conformation for the introduced residue. The quality of the obtained model was validated in PPW as described previously (Section 2.5), and the DROSHA mutated structures were subjected to 200 ns MD simulation.

### 2.8. Analysis of MD Simulation

The MD trajectory files were investigated by using simulation quality analysis (SQA) and simulation event analysis (SEA), along with simulation interaction diagram (SID) programs available with the Desmond module: root-mean-square deviation (RMSD), root-mean square fluctuation (RMSF), total intra-molecular hydrogen bonds, radius of gyration, along with secondary structure elements (SSE), were calculated and visualized. The recorded trajectories were clustered, and total energy values of the representative structures were calculated in Prime (options VSGV, OPLS3e)—the structures with minimal energy were compared and used for the following analysis of protein–RNA interactions (for structures of DROSHA complex with RNA). The binding strength and energetic properties between RNA and DROSHA protein were measured by molecular mechanics energies combined with the generalized Born and surface area continuum solvation (MM-GBSA) method [54] in Prime. The “MMGBSA dG Bind” parameter was recorded as the RNA-DROSHA binding free energy as calculated by the Prime Energy, a Molecular Mechanics + Implicit Solvent Energy Function (kcals/mol) = PrimeEnergy (Optimized Complex) − PrimeEnergy (Optimized Free miRNA) − PrimeEnergy (Optimized Free Protein).

## 3. Results

### 3.1. Assessment of Missense Mutations by Automated Prediction Tools

All available *DROSHA* variants acquired in hematologic and lymphoid tissues, consisting of 13 missense variants and 3 frameshift mutations, were subjected to the analysis. The consequences of frameshift variants were analyzed by the MutationTaster server (version 2, Charité–Universitätsmedizin Berlin, Berlin, Germany) [27]. P356Lfs*121 led to the loss of the most important protein features required for the main DROSHA functions: region (490–1374 aa) required for pri-miRNA processing activity and interaction with DGCR8, both RNAse III domains (876–1056 aa and 1107–1233 aa), downstream metal-binding sites, etc. A similar damaging effect was observed with R358Tfs*18. The consequences of mutation I1164Qfs*8 have an equal impact, even when it occurred at the C-terminal protein sequence: it affects the region (490–1374 aa) required for interaction with DGCR8 and pri-miRNA processing activity, metal-binding sites (1219, 1222 aa positions), and RNAase III domain 2 (1107–1233 aa).

**Figure 1:**
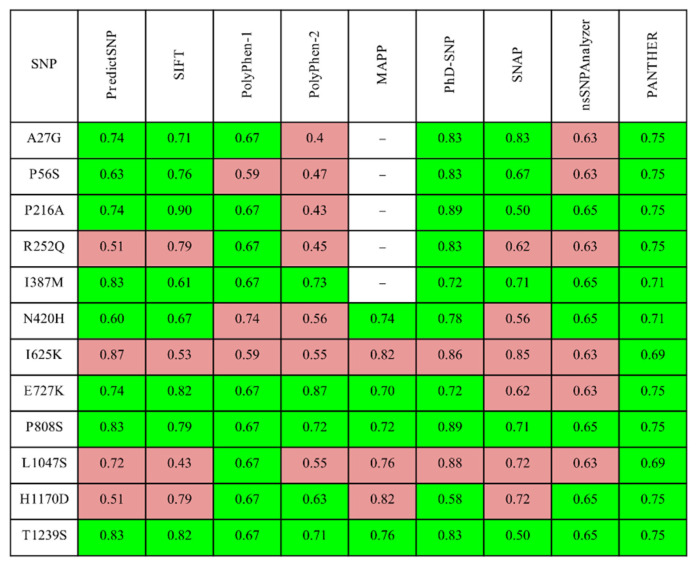
The expected accuracy results of the SNPs of *DROSHA* predicted as deleterious (marked in red) or benign (highlighted in green) in the PredictSNP server and integrated tools.

The functional effect of missense mutations in *DROSHA* was evaluated by several automated prediction tools, and the obtained results are presented in Figure 1. Based on this first level of analysis, R252Q, I625K, L1047S, and H1170D can be considered the most deleterious candidates. Additionally, the MutPred server predicted the probable molecular consequences of these selected mutations: I625K (altered DNA binding (Pr = 0.26 | *p* = 5.4 × 10^−3^), altered ordered interface (Pr = 0.24 | *p* = 0.04), gain of allosteric site at F623 (Pr = 0.21 | *p* = 0.03), altered stability (Pr = 0.14 | *p* = 0.02), loss of sulfation at Y627 (Pr = 0.02 | *p* = 0.02)), L1047S (altered ordered interface (Pr = 0.31 | *p* = 0.02), gain of allosteric site at F1044 (Pr = 0.28 | *p* = 3.9 × 10^−3^), altered metal binding (Pr = 0.16 | *p* = 0.03), gain of catalytic site at E1045 (Pr = 0.13 | *p* = 0.03), altered stability (Pr = 0.09 | *p* = 0.04)), and H1170D (altered metal binding (Pr = 0.51 | *p* = 3.6 × 10^−3^), gain of helix (Pr = 0.28 | *p* = 0.02)).

Four mutations had at least half of the predictions found to be damaging: R252Q, I625K, L1047S, and H1170D; therefore, they were used for the following analysis. Unfortunately, it was impossible to reconstruct the 252nd amino acid of DROSHA on the basis of available 3D-structures; therefore, R252Q was omitted from the structural analysis. The three remaining variants were analyzed by structure-based algorithms to predict the mutations’ effect on the protein stability by evaluating the vibrational entropy change and normal mode analysis. All the methods (Supplementary Table S1) predicted these variants to be destabilizing. The absolute value of resulting ΔΔG was more than 1.5 kcal/mol for I625K and L1047D variants, evaluated by the majority of algorithms considering these mutations to affect the structure along with protein functionality (Table 1). Indeed, the demonstrated effect is in compliance with the change in biochemical properties triggered by mutated amino acids: I625K—hydrophobic to positive, L1047S—hydrophobic to polar uncharged, and H1170D—positive to negatively charged.

### 3.2. Molecular Dynamics Simulation of DROSHA Protein

**Figure 2:**
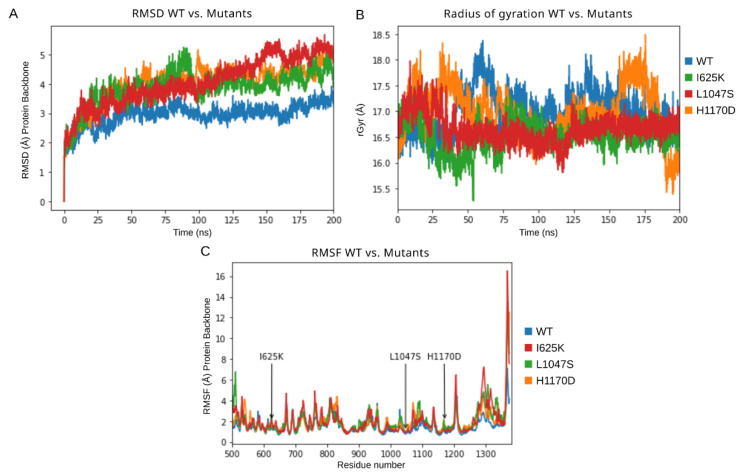
Results of 200 ns MD simulations of DROSHA wild-type protein along with its mutants (I625K, L1047S, H1170D). (**A**) RMSD trajectories of a protein’s backbone relative to the initial frame, (**B**) radius of gyration values, and (**C**) RMSF values of protein’s backbone.

In order to understand the detailed conformational changes in the protein due to these mutations, comparative MD simulations were carried out for 200 ns for each protein model. Primarily, the DROSHA protein structure was simulated without miRNA so as to study the mutations’ effect on the protein structure and stability independently. Various parameters have been analyzed throughout the simulation trajectory with a focus on RMSD compared to the initial frame (Figure 2A), RMSF (Figure 2C,D), energy parameters of the system (Table 2), total number of intramolecular hydrogen bonds (Table 2), radius of gyration (rGyr) (Table 2, Figure 2B), and structural secondary elements (Supplementary Figure S1) of the protein with the time-dependent function of MD.

The analysis of protein backbone RMSD compared to the initial frame demonstrated that the DROSHA wild-type protein obtained relative stability at an RMSD value of around 3 Å, whereas all the mutants showed a rising RMSD value up to 5 Å during the 200 ns simulation, not excluding the possibility of continued growth if the simulation was extended. Such a picture (Figure 2A) confirms the considerable destabilizing effect of these mutations on protein stability. RMSD results were in accordance with the calculated total energy of the studied proteins, which was remarkably higher for all mutants compared to wild-type DROSHA. From these results, L1047S substitution can be considered the most destabilizing among other examined mutations. The order of intra-protein hydrogen bonds (H-bonds) was calculated, and the resulted value represented a similar range with few more additional H-bonds for all associated mutants, as well as the radius of gyration can be considered common for all examined DROSHA structures. However, the fluctuation range of rGyr for I625K and L1047S mutants during MD simulation is noticeably slight (Figure 2B), which could be a signature of more rigid protein structures with less flexibility of particular elements and an increased tendency of compactness. RMSF fluctuations of each residue were also monitored during MD simulations, and the obtained plots represent the difference between wild-type DROSHA protein and its mutants (Figure 2C): the RMSF values of mutated structures were higher than those for native protein at the peaks corresponding to the introduced amino acid substitutions. The most considerable difference in RMSF values compared to native protein could be visualized at the C-terminus of the simulated protein structures: the RMSF peaks of mutated models at loci around the 1100th residue as well as the 1300th residue showed the regions with the most varying dynamics of obtained trajectories. Total energy of the systems with mutated proteins was observed to be higher compared to the wild-type DROSHA (Table 2).

**Figure 3:**
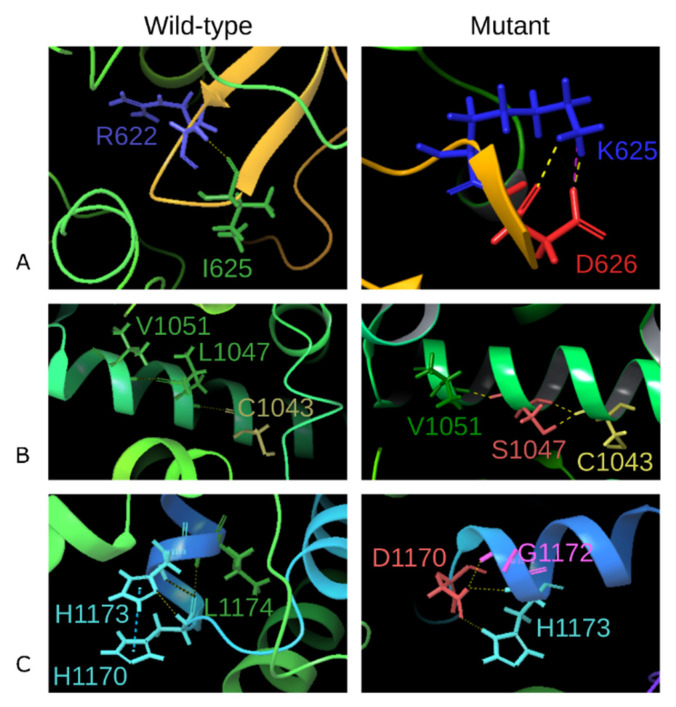
Comparison of interactions formed by the native and mutated protein residues in positions 625 (variant I625K, (**A**)), 1047 (L1047S, (**B**)), and 1170 (H1170D, (**C**)). The residue name and index are demonstrated. The yellow dashed line indicates H-bonds, salt-bridge is colored by magenta, and pi-pi stacking is shown by the blue dashed line.

The interactions and the corresponding bonds that were formed by the mutated residues were compared with the native DROSHA protein model. The 625th isoleucine residue forms a hydrogen bond with Arg622, fixing the β-sheet turn (Figure 3A), whereas the I625K mutant loses the foregoing H-bond and contacts with Asp626 through two H-bonds along with a newly formed salt-bridge, that indicates the loss of β-sheet stabilization and the formation of a very strong additional ionic interaction of the short bond between neighbor amino acids [55]. The 1047th leucine residue forms a single H-bond with Cys1043 and Val1051 within the α-helix, while the mutated Ser1047 creates an additional H-bond with Cys1043, which may indicate a less flexible helix formation (Figure 3B). Histidine at position 1170 interacts with histidine 1173 through the pi-pi stacking along with another H-bond, as well as contacts with L1174 via an H-bond. The pi-pi stacking is formed between H1170 at the α-helix and H1173 at the loop region, having the preferred interaction geometry of parallel–displaced arrangement—such fact suggests the stabilization of these elements relative to each other. Mutation H1170D leads to the loss of the pi-pi stacking interaction with H1173—only two H-bonds have remained with this residue. Additionally, a newly formed H-bond between D1170 and G1172 has occurred (Figure 3C).

### 3.3. Molecular Dynamics Simulations of DROSHA-miRNA Complex

**Figure 4:**
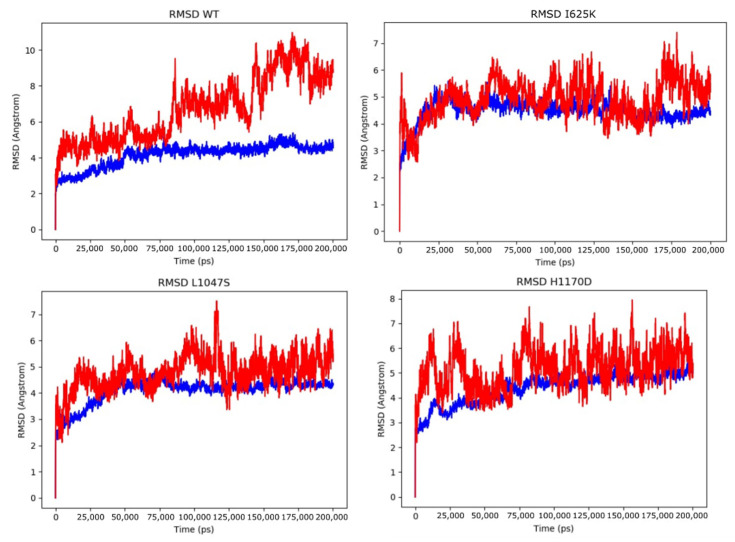
Results of 200 ns MD simulations of DROSHA wild-type protein along with its mutants (I625K, L1047S, H1170D) in a complex with miRNA. The RMSD plot for protein structure is colored blue, and RMSD fluctuations of miRNA are indicated by red.

The analogous MD simulations were performed for wild-type protein and its associated mutations in a complex with the miRNA molecule in the active site of DROSHA (PDB model 6V5B). In order to analyze the stability of the complex together with the dynamics behavior of RNA, the same parameters, such as RMSD and RMSF, were measured for DROSHA protein and RNA, along with H-bonds between them (Table 3) as well as the composition of secondary structural elements (Supplementary Figure S2). Analysis of H-bonds showed a similar range of interactions for wild-type and mutant structures. In contrast to the protein simulations of DROSHA alone, RMSD values of the native mutated proteins within the complex do not significantly differ from each other, indicating that the RNA molecule stabilizes the protein elements, offsetting the variants’ influence on the stability of the whole complex. However, the RMSD values of the RNA molecule within the native protein complex are noticeably higher than for mutant structures—this fact is supported by the miRNA stabilizing role of DGCR8 in the microprocessor complex [8], whereas DROSHA alone has weak binding capacity with miRNA. The RMSD fluctuations are presented on the corresponding plots in Figure 4. Moreover, the total energies of the complexes were calculated along with the binding free energy between protein and RNA (Table 4). Nevertheless, the calculated binding energy (ΔG) between protein and miRNA differs and varies from each introduced mutation: H1170D binds RNA weakly, while I625K and L1047S provide stronger interactions with miRNA, and L1047S is characterized by the most significant increase of binding capacity (Table 4). The obtained MD results are in compliance with the predictions of the mCMS-NA server (Table 1).

The interactions and corresponding molecular contacts of mutated residues within the DROSHA-RNA complex were analyzed and compared with the wild-type structure. The obtained results (Supplementary Figure S3) demonstrate that all residues under mutations lie in a close proximity to the miRNA binding site, although they do not interact directly with it. The same interactions were lost (pi-pi stacking of H1170D) and gained (salt-bridge by I625K) as those discussed for simulations of the protein alone. It is known that the DROSHA region 876–1056 aa corresponds to the first RNAase III domain (A), while the region of 1107–1233 aa is the second RNAase III domain (B) [56]. Therefore, both mutations, L1047S and H1170D, are located at the mentioned RNAase domains respectively, which specifies the direct as well as mediating impact of these substitutions on DROSHA functionality. Another mutation, I625K, does not appear to belong to the known functional domains. However, the mutagenesis of residues 622–623 demonstrated the abolishment of RNAase activity [56], and these residues are the closest neighbors of the analyzed I625K substitution; moreover, both I625 and K625 interact with Arg622 via H-bonds, suggesting that the substitution I625K also might indirectly affect the RNAase activity through local conformational shift. The summarized results of the DROSHA protein analysis are presented in Table 5.

## 4. Discussion

The significance of DROSHA in oncology is bound to its miRNA processing function, which is essential for miRNA biogenesis required for normal proliferation, among other processes in the cells [57]. Altered expression of miRNA processing genes was observed in several types of cancer [58], in particular in MDS patients [3]. Moreover, mutations in coding regions of *DROSHA* were also detected in several types of cancer, the majority of them acquired in the RNAase domains of the protein [59]. Recent research demonstrated the mutational burden of the *DROSHA* gene and the common significance of the miRNA processing pathway for MDS emergence [7]; therefore, the detailed investigation of *DROSHA* coding mutations is of primary importance.

All missense variants collected in hematological pathology were analyzed by various effect prediction tools, and three of the most damaging *DROSHA* mutations were indicated. The further detailed analysis of molecular dynamics simulation showed a compliance with the automated prognosis, and the main results can be summarized as follows: (1) the mutations were shown to be located in close proximity of the miRNA binding site, although mutated residues were not directly interacting with it, (2) all mutations were destabilizing for protein structure alone, however, they restrained the flexibility within the miRNA–protein complex, (3) all of the structures (protein alone as well as complex with miRNA) have demonstrated the increased total energy of the system for mutated structures, and (4) the local conformational shift was observed in all altered structures, triggered by the significant change of interaction partners and molecular bonds at the amino acid level.

Although the three considered mutations are rare events in oncohematology, the observed damaging effect indicates their distinct impact on DROSHA functionality and, therefore, on its endonuclease activity and consequent miRNA biosynthesis. We can hypothesize that even minor alterations in DROSHA activity might greatly affect the miRNA biogenesis and the generating content of miRNA, along with subsequent total dysregulation of protein expression during cancer progression. Supporting this statement, the resulting multiple miRNA expression changes were observed in MDS [60]. Our study showed that each of the three examined mutations incorporate a significant structural shift and rearrangement of bonds. The observed alterations can be considered as evoking certain clinical traits for oncohemathological disease progression, in particular MDS. Therefore, it appears that any coding mutations in *DROSHA* or its cellular partners of the same pathway should be extensively studied in each individual case to predict the impact of the lesion on the cancer progression and consequent phenotype.

## 5. Conclusions

The effect of *DROSHA* mutations found in oncohematological patients was analyzed via different computational tools. The three most deleterious *DROSHA* variants were determined and subjected to comprehensive analysis in molecular dynamics simulations. All of these variants were confirmed to be structurally destabilizing, with increased total energy of the system and consequent alterations in protein–miRNA binding. Particularly, I625K causes the new salt-bridge formation in the local protein loop, L1047S alters the H-bonding interaction within the alpha-helix, and H1170D leads to pi-pi stacking disruption. In summary, as oncohematological diseases and other clonal pathologies are characterized by dysregulated miRNA expression, any functional impairment of DROSHA, being the main component of the microprocessor complex, should be attentively investigated in patients with such malignancies in order to gain systematic comprehension of its role in pathogenesis.

## Figures and Tables

**Table 1:** Predicted stability changes (ΔΔG in kcal/mol) in DROSHA protein structure upon the selected mutations.

Variant	mCSM	mCMS- NA	MUpro	i- Mutant	SDM	DUET	PremPS	Maestro	FoldX4	Dyna Mut	Dyna Mut2
I625K	destab (−1.545)	stab (2.543)	destab (−2.234)	destab (−1.850)	destab (−1.080)	destab (−1.437)	destab (1.440)	destab (0.095)	destab (0.334)	stab (0.053)	destab (−1.42)
L1047S	destab (−3.220)	stab (3.590)	destab (−2.266)	destab (−1.520)	destab (−4.310)	destab (−3.578)	destab (2.460)	destab (0.614)	destab (4.975)	destab (−1.203)	destab (−3.10)
H1170D	destab (−0.837)	destab (−4.107)	destab (−0.539)	destab (−0.70)	destab (−0.660)	destab (−0.812)	destab (1.28)	destab (0.254)	destab (1.325)	destab (−0.216)	destab (−0.45)

“Destab” indicates the decrease of stability, and “stab” corresponds to the increase. sCMS-NA column presents the Gibbs energy changes for affinity with RNA.

**Table 2:** Statistics of MD simulations. Intra-protein H-bonds, radius of gyration, RMSD values, total energy of the protein, and mean energy of clustered frames are presented.

Model	H-Bonds Intra		rGyr (Å)		RMSD Backbone (Å)		Total Energy (kcal/mol)
	Range	Mean	Range	Mean	Range	Mean	Mean
Drosha wt	(708, 803)	754.339	(15.979, 18.376)	16.995	(0.000, 3.930)	2.964	−33,438
I625K	(720, 821)	770.246	(15.257, 17.527)	16.523	(0.000, 5.273)	3.871	−32,940
L1047S	(715, 817)	761.297	(15.81, 17.982)	16.666	(0.000, 5.689)	4.077	−32,897
H1170	(729, 833)	774.806	(15.385, 18.494)	16.975	(0.000, 5.284)	3.982	−33,049

**Table 3:** Statistics of MD simulations of DROSHA complex with RNA. H-bonds between protein and RNA and RMSD values are presented.

Model	H-Bonds RNA–Protein		RMSD Protein (Å)			RMSD RNA (Å)		
	Range	Mean	Range	Mean	SD	Range	Mean	SD
Drosha WT	(28, 64)	47.141	(0.000, 5.380)	4.146	0.634	(0.000, 10.969)	6.697	1.803
H1170	(30, 67)	45.527	(0.000, 5.556)	4.388	0.619	(0.000, 7.947)	5.151	0.807
I625K	(33, 66)	46.066	(0.000, 5.588)	4.475	0.417	(0.000, 7.396)	4.893	0.710
L1047S	(32, 73)	47.392	(0.000, 4.852)	4.099	0.468	(0.000, 7.511)	4.774	0.674

**Table 4:** Statistics of energy values of DROSHA complex with RNA. Minimal total energy and mean total energy values are presented. Binding energy (ΔG Bind) between protein and RNA was calculated by the MM-GBSA method.

Model	Minimal Total Energy of the Complex (kcal/mol)	Mean Total Energy of the Complex (kcal/mol)	MM-GBSA ΔG Bind (kcal/mol)
Drosha WT	−46,193.114	−46,049.497	−380.929
I625K	−45,830.501	−45,711.641	−386.547
L1047S	−45,787.443	−45,687.955	−439.187
H1170D	−45,941.373	−45,869.388	−334.761

**Table 5:** Summary of I625K, L1047S, and H1170D effect evaluation. Histology-based diagnosis was obtained from the COSMIC database. Functional domains were described in Kwon et al., 2016. Shifts in backbone RMSD and corresponding energies are calculated as follows: *value_wild-type_–value_mutant_*.

Mutation	Histology-Based Diagnosis	Functional Domains	Sequence-Based Tools Predicting Functional Impairment	Structure-Based Tools Predicting Molecular Destabilization	ΔRMSD Backbone (Free Protein, Å)	ΔG (Free Protein, kcal/mol)	ΔG (miRNA Protein Complex, kcal/mol)	ΔG Bind (miRNA, kcal/mol)
I625K	Adult T cell lymphoma-leukemia	Central domain (Platform)	8/9	9/11	0.91	498 (destabilization)	337.86 (destabilization)	−5.62 (increased affinity)
L1047S	B cell prolymphocytic leukemia	RNAse IIIa	7/9	10/11	1.11	541 (destabilization)	361.54 (destabilization)	−58.26 (increased affinity)
H1170D	Breast implant-associated anaplastic large cell lymphoma, invasive	RNAse IIIb	4/9	11/11	1.02	389 (destabilization)	180.11 (destabilization)	46.17 (decreased affinity)

## Supplementary Materials

The following are available online at https://www.mdpi.com/article/10.3390/cells10092357/s1. Figure S1: Comparison of DROSHA residues SSE through the MD simulation, Figure S2: Comparison of residues SSE through the MD simulation of DROSHA protein in a complex with miRNA, Figure S3: Comparison of interactions formed by the native and mutated residues in DROSHA complex with miRNA, Table S1: The prediction methods used to evaluate structural and functional consequences of the analyzed DROSHA variants.

## References

[1] Steensma D.P., Bejar R., Jaiswal S., Lindsley R.C., Sekeres M., Hasserjian R.P., Ebert B.L. (2015). Clonal hematopoiesis of indeterminate potential and its distinction from myelodysplastic syndromes. Blood.

[2] Meggendorfer M., Haferlach C., Kern W., Haferlach T. (2017). Molecular analysis of myelodysplastic syndrome with isolated deletion of the long arm of chromosome 5 reveals a specific spectrum of molecular mutations with prognostic impact: A study on 123 patients and 27 genes. Haematologica.

[3] Santamaria C., Muntion S., Roson B., Blanco B., Lopez-Villar O., Carrancio S., Sanchez-Guijo F.M., Diez-Campelo M., Alvarez-Fernandez S., Sarasquete M.E. (2012). Impaired expression of Dicer, Drosha, SBDS and some microRNAs in mesenchymal stromal cells from myelodysplastic syndrome patients. Haematologica.

[4] Rhyasen G.W., Starczynowski D.T. (2011). Deregulation of microRNAs in myelodysplastic syndrome. Leukemia.

[5] Meunier M., Guttin A., Ancelet S., Laurin D., Zannoni J., Lefebvre C., Tondeur S., Persoons V., Pezet M., Pernet-Gallay K. (2020). Extracellular vesicles from myelodysplastic mesenchymal stromal cells induce DNA damage and mutagenesis of hematopoietic stem cells through miRNA transfer. Leukemia.

[6] Raaijmakers M.H.G.P., Mukherjee S., Guo S., Zhang S., Kobayashi T., Schoonmaker J.A., Ebert B.L., Al-Shahrour F., Hasserjian R.P., Scadden E.O. (2010). Bone progenitor dysfunction induces myelodysplasia and secondary leukemia. Nature.

[7] Moiseev I.S., Tcvetkov N.Y., Barkhatov I.M., Barabanshikova M.V., Bug D.S., Petuhova N.V., Tishkov A.V., Bakin E.A., Izmailova E.A., Shakirova A.I. (2021). High mutation burden in the checkpoint and micro-RNA processing genes in myelodysplastic syndrome. PLoS ONE.

[8] Li S., Patel D.J. (2016). Drosha and Dicer: Slicers cut from the same cloth. Cell Res..

[9] Lee Y., Ahn C., Han J., Choi H., Kim J., Yim J., Lee J., Provost P., Rådmark O., Kim S. (2003). The nuclear RNase III Drosha initiates microRNA processing. Nature.

[10] Morin R., Assouline S., Alcaide M., Mohajeri A., Johnston R., Chong L., Grewal J., Yu S., Fornika D., Bushell K. (2015). Genetic landscapes of relapsed and refractory diffuse large B-cell lymphomas. Clin. Cancer Res..

[11] Nangalia J., Massie C., Baxter E.J., Nice F., Gundem G., Wedge D., Avezov E., Li J., Kollmann K., Kent D. (2013). Somatic CALR mutations in myeloproliferative neoplasms with nonmutated JAK2. N. Engl. J. Med..

[12] Jiang L., Gu Z.-H., Yan Z.-X., Zhao X., Xie Y.-Y., Zhang Z.-G., Pan C.-M., Hu Y., Cai C.-P., Dong Y. (2015). Exome sequencing identifies somatic mutations of DDX3X in natural killer/T-cell lymphoma. Nat. Genet..

[13] Gunawardana J., Chan F.C., Telenius A., Woolcock B., Kridel R., Tan K.L., Ben-Neriah S., Mottok A., Lim R., Boyle M. (2014). Recurrent somatic mutations of PTPN1 in primary mediastinal B cell lymphoma and Hodgkin lymphoma. Nat. Genet..

[14] Kataoka K., Nagata Y., Kitanaka A., Shiraishi Y., Shimamura T., Yasunaga J.-I., Totoki Y., Chiba K., Sato-Otsubo A., Nagae G. (2015). Integrated molecular analysis of adult T-cell leukemia/lymphoma. Nat. Genet..

[15] Beà S., Valdés-Mas R., Navarro A., Salaverria I., Garcia D.M., Jares P., Giné E., Pinyol M., Royo C., Nadeu F. (2013). Landscape of somatic mutations and clonal evolution in mantle cell lymphoma. Proc. Natl. Acad. Sci. USA.

[16] Chapiro E., Pramil E., Diop M., Roos-Weil D., Dillard C., Gabillaud C., Maloum K., Settegrana C., Baseggio L., Lesesve J.-F. (2019). Genetic characterization of B-cell prolymphocytic leukemia: A prognostic model involving MYC and TP53. Blood.

[17] Laurent C., Nicolae A., Laurent C., Le Bras F., Haioun C., Fataccioli V., Amara N., Adélaïde J., Guille A., Schiano J.-M. (2019). Gene alterations in epigenetic modifiers and JAK-STAT signaling are frequent in breast implant-associated ALCL. Blood.

[18] Sim N.-L., Kumar P., Hu J., Henikoff S., Schneider G., Ng P.C. (2012). SIFT web server: Predicting effects of amino acid substitutions on proteins. Nucleic Acids Res..

[19] Adzhubei I., Jordan D., Sunyaev S.R. (2013). Predicting functional effect of human missense mutations using PolyPhen-2. Curr. Protoc. Hum. Genet..

[20] Li B., Krishnan V.G., Mort M.E., Xin F., Kamati K.K., Cooper D.N., Mooney S.D., Radivojac P. (2009). Automated inference of molecular mechanisms of disease from amino acid substitutions. Bioinformatics.

[21] Choi Y., Chan A.P. (2015). PROVEAN web server: A tool to predict the functional effect of amino acid substitutions and indels. Bioinformatics.

[22] Bao L., Zhou M., Cui Y. (2005). nsSNPAnalyzer: Identifying disease-associated nonsynonymous single nucleotide polymorphisms. Nucleic Acids Res..

[23] Stone E.A. (2005). Physicochemical constraint violation by missense substitutions mediates impairment of protein function and disease severity. Genome Res..

[24] Capriotti E., Calabrese R., Casadio R. (2006). Predicting the insurgence of human genetic diseases associated to single point protein mutations with support vector machines and evolutionary information. Bioinformatics.

[25] Bromberg Y., Yachdav G., Rost B. (2008). SNAP predicts effect of mutations on protein function. Bioinformatics.

[26] Tang H., Thomas P. (2016). PANTHER-PSEP: Predicting disease-causing genetic variants using position-specific evolutionary preservation. Bioinformatics.

[27] Schwarz J.M., Cooper D.N., Schuelke M., Seelow D. (2014). MutationTaster2: Mutation prediction for the deep-sequencing age. Nat. Methods.

[28] Bendl J., Stourac J., Salanda O., Pavelka A., Wieben E.D., Zendulka J., Brezovsky J., Damborsky J. (2014). PredictSNP: Robust and accurate consensus classifier for prediction of disease-related mutations. PLoS Comput. Biol..

[29] Pires D.E.V., Ascher D., Blundell T.L. (2013). mCSM: Predicting the effects of mutations in proteins using graph-based signatures. Bioinformatics.

[30] Cheng J., Randall A., Baldi P. (2005). Prediction of protein stability changes for single-site mutations using support vector machines. Proteins Struct. Funct. Bioinform..

[31] Capriotti E., Fariselli P., Casadio R. (2005). I-Mutant2.0: Predicting stability changes upon mutation from the protein sequence or structure. Nucleic Acids Res..

[32] Worth C., Preissner R., Blundell T.L. (2011). SDM—A server for predicting effects of mutations on protein stability and malfunction. Nucleic Acids Res..

[33] Pires D.E., Ascher D., Blundell T.L. (2014). DUET: A server for predicting effects of mutations on protein stability using an integrated computational approach. Nucleic Acids Res..

[34] Chen Y., Lu H., Zhang N., Zhu Z., Wang S., Li M. (2020). PremPS: Predicting the impact of missense mutations on protein stability. PLoS Comput. Biol..

[35] Laimer J., Hofer H., Fritz M., Wegenkittl S., Lackner P., Laimer J., Hofer H., Fritz M., Wegenkittl S., Lackner P. (2015). MAESTRO—Multi agent stability prediction upon point mutations. BMC Bioinform..

[36] Pires D.E.V., Ascher D.B. (2017). mCSM-NA: Predicting the effects of mutations on protein–nucleic acids interactions. Nucleic Acids Res..

[37] Tokuriki N., Tawfik D.S. (2009). Stability effects of mutations and protein evolvability. Curr. Opin. Struct. Biol..

[38] Calloni G., Zoffoli S., Stefani M., Dobson C.M., Chiti F. (2005). Investigating the effects of mutations on protein aggregation in the cell. J. Biol. Chem..

[39] Randles L.G., Lappalainen I., Fowler S.B., Moore B., Hamill S.J., Clarke J. (2006). Using model proteins to quantify the effects of pathogenic mutations in Ig-like proteins. J. Biol. Chem..

[40] Schymkowitz J., Borg J., Stricher F., Nys R., Rousseau F., Serrano L. (2005). The FoldX web server: An online force field. Nucleic Acids Res..

[41] Frappier V., Chartier M., Najmanovich R.J. (2015). ENCoM server: Exploring protein conformational space and the effect of mutations on protein function and stability. Nucleic Acids Res..

[42] Rodrigues C.H.M., Pires D.E.V., Ascher D.B. (2018). DynaMut: Predicting the impact of mutations on protein conformation, flexibility and stability. Nucleic Acids Res..

[43] Rodrigues C.H., Pires D.E., Ascher D.B. (2020). DynaMut2: Assessing changes in stability and flexibility upon single and multiple point missense mutations. Protein Sci..

[44] Partin A.C., Zhang K., Jeong B.-C., Herrell E., Li S., Chiu W., Nam Y. (2020). Cryo-EM structures of human Drosha and DGCR8 in complex with primary microRNA. Mol. Cell.

[45] Berman H.M., Westbrook J.D., Feng Z., Gilliland G., Bhat T.N., Weissig H., Shindyalov I.N., Bourne P.E. (2000). The protein data bank. Nucleic Acids Res..

[46] Jacobson M.P., Pincus D.L., Rapp C.S., Day T.J., Honig B., Shaw D.E., Friesner R.A. (2004). A hierarchical approach to all-atom protein loop prediction. Proteins: Struct. Funct. Bioinform..

[47] Li J., Abel R., Zhu K., Cao Y., Zhao S., Friesner R.A. (2011). The VSGB 2.0 model: A next generation energy model for high resolution protein structure modeling. Proteins: Struct. Funct. Bioinform..

[48] Harder E., Damm W., Maple J., Wu C., Reboul M., Xiang J.Y., Wang L., Lupyan D., Dahlgren M.K., Knight J.L. (2015). OPLS3: A force field providing broad coverage of drug-like small molecules and proteins. J. Chem. Theory Comput..

[49] Roos K., Wu C., Damm W., Reboul M., Stevenson J.M., Lu C., Dahlgren M.K., Mondal S., Chen W., Wang L. (2019). OPLS3e: Extending force field coverage for drug-like small molecules. J. Chem. Theory Comput..

[50] Sastry G.M., Adzhigirey M., Day T., Annabhimoju R., Sherman W. (2013). Protein and ligand preparation: Parameters, protocols, and influence on virtual screening enrichments. J. Comput. Mol. Des..

[51] Bowers K.J., Chow D.E., Xu H., Dror R.O., Eastwood M.P., Gregersen B.A., Klepeis J.L., Kolossvary I., Moraes M.A., Sacerdoti F.D. Scalable algorithms for molecular dynamics simulations on commodity clusters. Proceedings of the 2006 ACM/IEEE Conference on Supercomputing.

[52] Jorgensen W.L., Chandrasekhar J., Madura J., Impey R.W., Klein M.L. (1983). Comparison of simple potential functions for simulating liquid water. J. Chem. Phys..

[53] Nosé S. (1984). A unified formulation of the constant temperature molecular dynamics methods. J. Chem. Phys..

[54] Genheden S., Ryde U. (2015). The MM/PBSA and MM/GBSA methods to estimate ligand-binding affinities. Expert Opin. Drug Discov..

[55] Bissantz C., Kuhn B., Stahl M. (2010). A medicinal chemist’s guide to molecular interactions. J. Med. Chem..

[56] Kwon S.C., Nguyen T.A., Choi Y.-G., Jo M.H., Hohng S., Kim V.N., Woo J.-S. (2015). Structure of human Drosha. Cell.

[57] Gurtner A., Falcone E., Garibaldi F., Piaggio G. (2016). Dysregulation of microRNA biogenesis in cancer: The impact of mutant p53 on Drosha complex activity. J. Exp. Clin. Cancer Res..

[58] Yan M., Huang H.-Y., Wang T., Wan Y., Cui S.-D., Liu Z.-Z., Fan Q.-X. (2011). Dysregulated expression of Dicer and Drosha in breast cancer. Pathol. Oncol. Res..

[59] Hata A., Kashima R. (2015). Dysregulation of microRNA biogenesis machinery in cancer. Crit. Rev. Biochem. Mol. Biol..

[60] Kuang X., Chi J., Wang L. (2016). Deregulated microRNA expression and its pathogenetic implications for myelodysplastic syndromes. Hematology.

