# Supplementary material for: Towards Understanding the Pathogenicity of DROSHA Mutations in Oncohematology
Source: Cells. 2021 Sep 8;10(9):2357. doi: 10.3390/cells10092357 (PMC8471307; doi:10.3390/cells10092357)
Supplement: Supplementary file 1 [file cells-10-02357-s001.zip › cells-1363676-supplementary.pdf]

cells

MDPI

*Communication*

# Towards Understanding the Pathogenicity of DROSHA Mutations in Oncohematology

**Dmitrii S. Bug [1], Artem V. Tishkov [1], Ivan S. Moiseev [2], Yuri B. Porozov [3,4,*] and Natalia V. Petukhova [1,*]**

## Supplementary Materials

**Table S1.** The prediction methods used to evaluate structural and functional consequences of the analysed DROSHA variants.

| Tool name | Prediction algorithm description | Reference |
|---|---|---|
| SIFT | Sorting Intolerant from Tolerant (SIFT) algorithm provides prediction based on the degree of conservation of amino acid residues in sequence alignments. | Sim et al., 2012 |
| PolyPhen-2 | Polymorphism phenotyping-2 server predicts possible effect building conservation profile based on the probability of the missense mutation being damaging for gene transcripts, protein sequence annotations and structural attributes. | Adzhubei et al., 2013 |
| MutPred | MutPred tool is based upon protein sequence and models changes of structural features and functional sites between wild-type and mutant sequences utilizing a random forest algorithm with data based upon the probabilities of loss or gain of properties. | Li et al., 2009 |
| nsSNPAnalyzer | nsSNPAnalyzer server integrates multiple sequences alignment and protein structure analysis to identify disease-associated nsSNPs applying machine learning Random Forest method to predict the nsSNP's effect from structural and evolutionary information of the query. | Bao et al., 2005 |
| MAPP | The algorithm calculates the physicochemical characteristics in each position of the protein, based on observed evolutionary variation. | Stone, 2005 |
| PhD-SNP | PhD-SNP is a binary classifier based on a Gradient Boosting algorithm estimating sequence-based features and their conservation score. | Capriotti et al., 2006 |
| SNAP | Method SNAP (screening for non-acceptable polymorphisms) predicts the functional effects of single amino acid substitutions comdining many sequence analysis tools in a battery of neural networks to predict the functional effects of nsSNPs. | Bromberg et al., 2008 |

| | | |
|---|---|---|
| PANTHER | The algorithm employs prediction based on 'evolutionary preservation': homologous proteins are used to reconstruct the likely sequences of ancestral proteins at nodes in a phylogenetic tree, and the direct association between the conservation time and the likelihood of functional impact is calculated. | Tang & Thomas, 2016 |
| PredictSNP | The server combines the prediction of several tools and represents the consensus classifier pointing to the prioritized estimation. | Bendl et al., 2014 |
| MutationTaster | The server evaluates the pathogenicity of potential amino acid substitutions, intronic and synonymous alterations, indels based on the information derived from various biomedical databases and established analysis programs. | Schwarz et al., 2014 |
| mCMS | mCSM is a machine learning method to predict the effects of missense mutations based on structural signatures derived from the graph-based concept of Cutoff Scanning Matrix (CSM). | Pires et al., 2014 |
| SDM | The SDM approach compares amino acid propensities for the wild-type and mutant proteins in the folded and unfolded states in order to estimate the free energy differences between wild type and mutant based on statistical potential energy function that uses environment-specific amino-acid substitution frequencies within homologous protein families to calculate a stability score. | Worth et al., 2011 |
| MUpro | MUpro uses support vector machines to predict protein stability changes for single amino acid mutations leveraging both sequence and structural information. | Cheng et al., 2005 |
| i-Mutant | i-Mutant is a support vector machine tool for the automatic prediction of protein stability changes upon single point mutations based on structure and sequence information of the query trained on thermodynamic experimental data of free energy. | Capriotti et al., 2005 |
| DUET | DUET server is an integrated tool combining two mCMS and SDM approaches and generating a consensus prediction, obtained by combining the results of the separate methods in an optimized predictor using Support Vector Machines. | Pires et al., 2014 |
| PremPS | PremPS tool estimates the effect of mutation in protein stability which is composed of ten evolutionary- and structure-based features and parameterized on a balanced dataset with an equal number of stabilizing and destabilizing mutations. | Chen et al., 2020 |

| | | |
|---|---|---|
| Maestro | Maestro is a structure-based method for protein stability prediction which implements a multi-agent machine learning system and provides a predicted free energy change. | Laimer et al., 2015 |
| mCMS-NA | mCMS-NA is a graph-based method quantitatively predicting the effects of mutations in protein coding regions on nucleic acid binding affinities. | Pires & Ascher, 2017 |
| FoldX4 | FoldX is an empirical force field based on empirical effective energy functions which calculates the free energy and evaluates the effect of mutations on the stability, folding and dynamics of proteins and nucleic acids. | Schymkowitz et al., 2005 |
| ENCoM | ENCoM is a coarse-grained normal mode analysis method to predict the effect of single point mutations on protein dynamics and thermostability resulting from vibrational entropy changes. | Frappier et al., 2015 |
| DynaMut<br>DynaMut2 | DynaMut is a consensus predictor implementing ENCoM and sCMS normal mode approaches. DynaMut2, a web server that combines Normal Mode Analysis (NMA) methods to capture protein motion and our graph-based signatures to represent the wildtype environment to investigate the effects of single and multiple point mutations on protein stability and dynamics | Rodrigues et al., 2018<br>Rodrigues et al., 2021 |

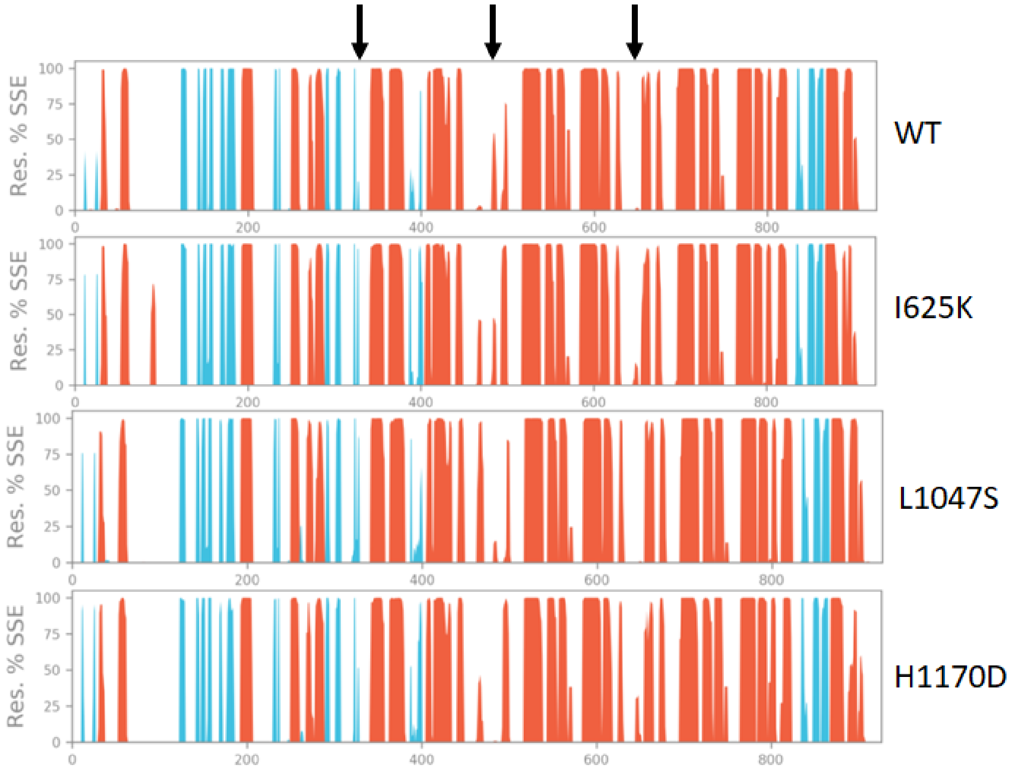


**A** .

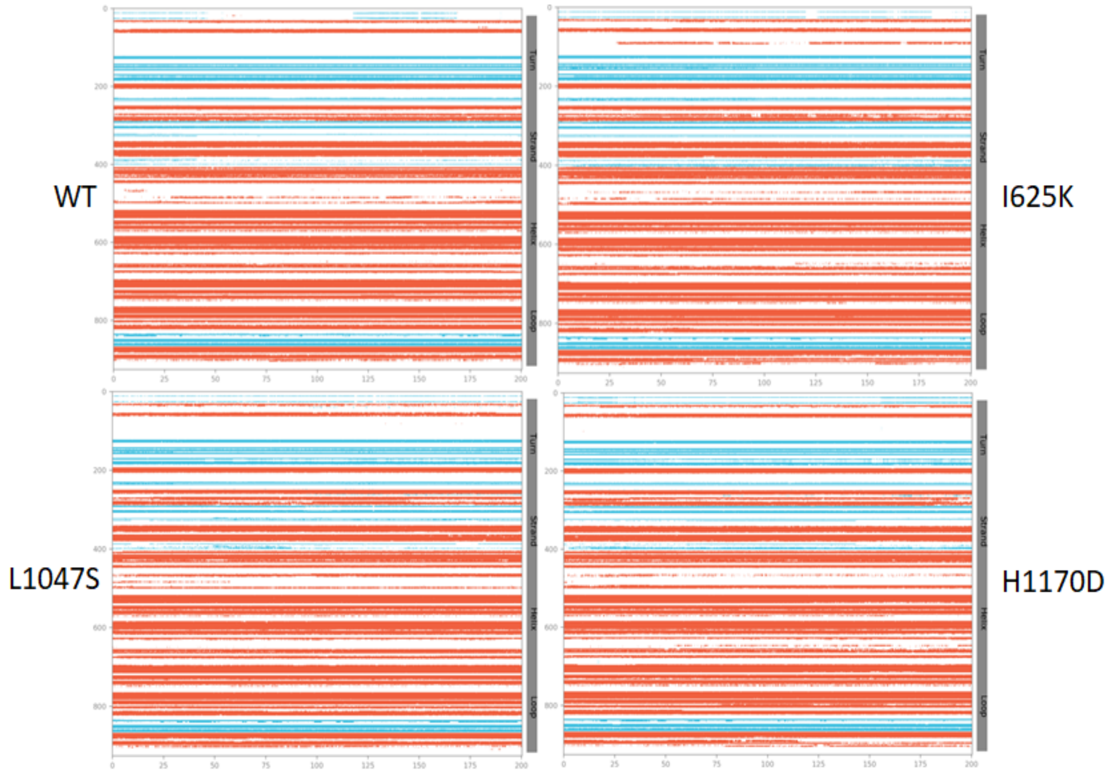


**B**

**Figure S1. Comparison of DROSHA residues SSE through the MD simulation.** (**A**) SSE distribution by residue index throughout the protein structures. Arrows indicate the discordancies in SSE compared to wild-type protein and mutants. (**B**) Monitoring of each residue and its SSE assignment over time.The residues index do not correspond to the positions in the protein sequence as the initial model starts from the 441the aa with one loop unresolved [463-500 aa] (see Methods section), therefore the indexes on the picture up to 100th can be omitted. $\alpha$-Helices are indicated in red, β-sheets are coloured by blue.

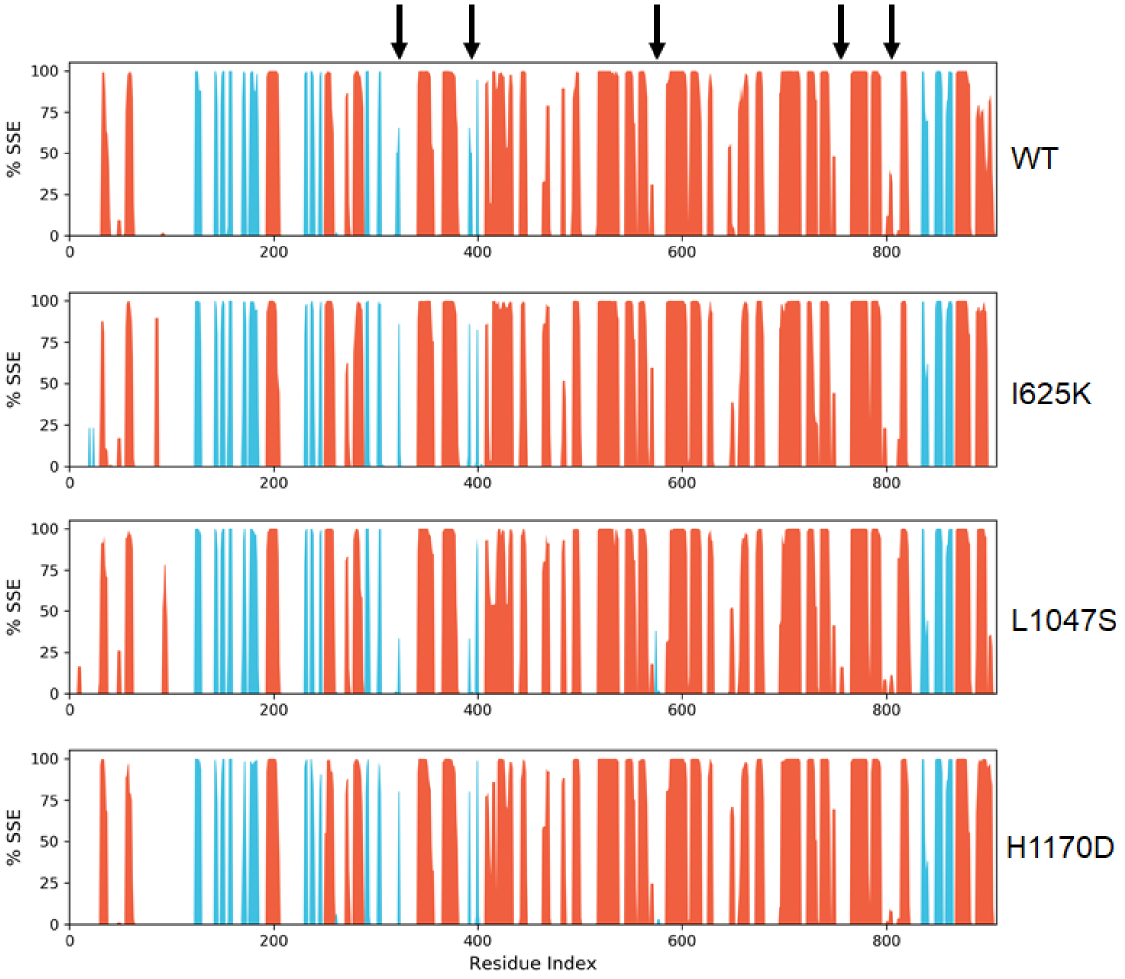


**Figure S2. Comparison of residues SSE through the MD simulation of DROSHA protein in a complex with miRNA.** SSE distribution by residue index throughout the protein structures. Arrows indicate the discordancies in SSE compared to wild-type protein and mutants. The residues index do not correspond to the positions in the protein sequence as the initial model starts from the 441th aa with one loop unresolved [463-500 aa] (see Methods section), therefore the indexes on the picture up to 100th can be omitted. $\alpha$-Helices are indicated in red, $\beta$-sheets are coloured by blue.

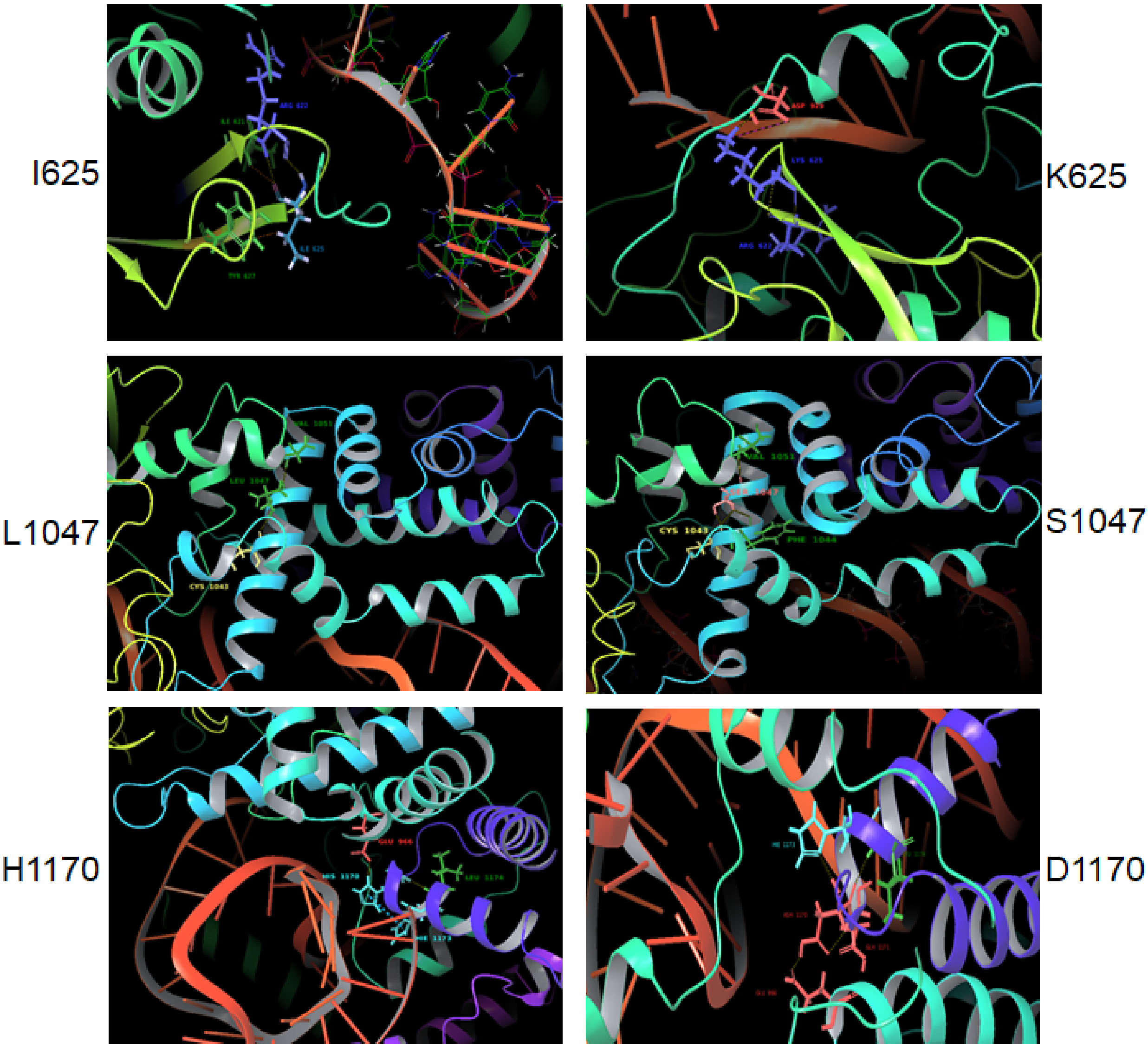


**Figure S3. Comparison of interactions formed by the native and mutated residues in DROSHA complex with miRNA.** The residue name and index are demonstrated. The yellow dash line indicates H-bonds, salt-bridge is colored by magenta, pi-pi stacking is shown by the blue dash line, and clushes contacts - by orange ones. miRNA is presented as a red colored structure.
